# Comparison of cardiorespiratory fitness between preschool children with normal and excess body adipose ~ An observational study

**DOI:** 10.1371/journal.pone.0223907

**Published:** 2019-10-11

**Authors:** Sheng-Hui Tuan, Chien-Hui Li, Shu-Fen Sun, Min-Hui Li, I-Hsiu Liou, Tzu-Ping Weng, I-Hsuan Chen, Ko-Long Lin

**Affiliations:** 1 Department of Rehabilitation Medicine, Cishan Hospital, Ministry of Health and Welfare, Cishan District, Kaohsiung City, Taiwan (R.O.C.); 2 Department of Physical Therapy, Shu-Zen junior College of Medicine and Management, Luzhu Dist., Kaohsiung City, Taiwan (R.O.C.); 3 Department of Physical Medicine and Rehabilitation, Kaohsiung Veterans General Hospital, Zuoying Dist., Kaohsiung City, Taiwan (R.O.C.); 4 Department of Physical Therapy, Foo-Yin University, Daliao Dist., Kaohsiung City, Taiwan (R.O.C.); University of Cádiz, SPAIN

## Abstract

**Objective:**

Overweight and obesity in preschoolers might develop into childhood and even adulthood obesity. Overweight and obesity have been shown to be negatively related with cardiorespiratory fitness (CRF) in children and adults but few studies did among preschoolers. We aimed to evaluate whether excess body adipose is negatively associated with CRF in both the submaximal and maximal effort of preschool children in exercise testing and to examine if there is difference to achieve maximal effort during exercise testing between preschoolers with normal and excess body adipose.

**Methods:**

Data of 106 preschoolers aged 4–6 that received symptom-limited treadmill exercise testing was analyzed. Anthropometry was measured by vector bioelectrical impedance analysis. Excess body adipose was defined as (1) ‘overweight’ and ‘obesity’ by body mass index (BMI), (2) fat mass index (FMI) greater than the sex- and age-specific 75th percentile of whole subjects, and (3) fat-free mass index (FFMI) smaller than the sex- and age-specific 25th percentile. CRF was indicated by metabolic equivalent (MET) at anaerobic threshold (AT MET), peak MET, oxygen uptake efficiency slope (OUES) calculated by the 50% (OUES-50) and the entire (OUES-100) duration of the exercise testing.

**Results:**

Preschoolers with excess body adipose by three different definitions (BMI, FMI, and FFMI) all had poorer ability to perform maximal effort (p = 0.004, 0.043, and 0.007, respectively). Preschoolers with excess body adipose by BMI and FFMI classifications had lower OUES-50 (p = 0.018, and 0.001, respectively), and lower OUES-100 (p = 0.004, and 0.001, respectively) than peers with normal body adipose during exercise testing while those with excess body adipose by FMI classification showed no significant differences from peers with normal body adipose in both OUES-50 and OUES-100.

**Conclusions:**

Preschoolers with excess body adipose had lower CRF significantly during treadmill exercise testing. Weight control and health promotion should start as early as possible.

## Introduction

More and more evidences suggest a dramatic increase in prevalence of childhood obesity over the past few decades and it is now recognized as a serious public health concern[1, 2]. Based on a nationwide survey by Ministry of Education of Taiwan in 2003, the prevalence of overweight and obesity was 15.2% among school girls and 25.2% among school boys[3]. Furthermore, the increase of the prevalence has been documented as early as the preschool years. In one worldwide study, De Onis et al. showed that the global prevalence of preschool overweight and obesity has escalated from 4.2% in 1990 to 6.7% in 2010, with a projected increase to 9.1% in 2020[2]. Preschool obesity might cause serious health consequences in both physical and psychological aspects later in life[4]. It increase early risk for much of adult morbidity, such as type 2 diabetes[5], hypertension and dyslipidemia, cardiovascular disease[6], asthma and sleep apnea[7], and even premature death[8]. It is also associated with lower self-esteem, psychological and social stress[9]. Moreover, overweight and obesity in childhood will track into adulthood and is difficult to treat successfully[10].

More and more strategies for the prevention of overweight and obesity in preschool children have developed in recent years and many of them focus on increases in physical activity (PA) [11] since an active lifestyle in children has been linked to positive health outcomes such smaller waist circumference and lower body mass index (BMI), cardiovascular indicators and bone health[12, 13]. Overweight and obesity have been shown to be negatively related with cardiorespiratory fitness (CRF) level in school children and adolescents [14, 15]. However, BMI reflects both the fat mass (FM) and the fat-free mass (FFM) in the body, which may have different associations with physical fitness[16], and it had been proved to correlate poorly with % fat mass (%FM) in a preschool population[17]. Based on currently presented few studies, FM index (FMI) and FFM index (FFMI) might be better than BMI in reflecting the relationship between body adipose and CRF in preschool children[18, 19].

CRF is defined as the overall capacity of the cardiovascular and respiratory systems to carry out prolonged strenuous exercise. Cardiopulmonary exercise testing (CPET) is the gold standard to measure CRF. A limited number of studies have determined the CRF of preschoolers by CPET given the smaller body size relative to the testing equipment, the inability to sustain activity to a maximal level, the reduced motivation, and the potentially shorter attention span[20]. The oxygen uptake efficiency slope (OUES) estimates the ventilatory efficiency with respect to oxygen consumption (VO_2_) [21]. It is now a well-established substitute for maximal VO_2_ (VO_2_ max)in submaximal exercise effort among adults and older children[22], and our study proved that it could be also used in preschool children[19] since they may not be able to sustain activity to maximal level during CPET. To our knowledge, no available studies evaluate the association between excess body adipose, defined by BMI, FMI, or FFMI, and the CRF indicated by OUES or maximal VO_2_ during CPET, among preschool children. We aimed to evaluate whether excess body adipose is negatively associated with CRF (as indicated by peak VO2 in the maximal effort and OUES in the submaximal effort) of preschool children measured directly from CPET. We also examined if there is difference to achieve maximal effort during exercise testing between preschoolers with normal and excess body adipose in this study.

## Methods

### Subject characteristics

This was a retrospective cross sectional study. The participants in this study included preschool children aged from 4 to 6 years old. All the recruited preschool children took CPET at a tertiary medical center in the southern Taiwan, from February 2012 to February 2017. The inclusion criteria were as follows: preschoolers who (1) completed transthoracic echocardiographic examination and 12-lead electrocardiogram without known significant medical conditions and without detectable cardiovascular diseases, (2) understood the steps of CPET, and (3) showed no signs of acute infection or fever 3 days prior to CPET. All the participants received body composition measurement first and then CPET on the same day. We used G*Power software (version 3.1.9.2, for Windows)[23] to determine minimum sample size. For comparisons of CRF between preschoolers with excess and normal body adipose under different classifications, the minimum sample size was 64 in preschoolers with normal adipose group and 16 in preschoolers with excessive adipose group with type 1 error = 0.05, power = 0.8, large effect size, and allocation ratio = 4 (based on a nationwide survey by Ministry of Education of Taiwan in 2003[3]). The mean (M) and standard deviation (SD) for effect size estimation were based on the study we conducted before in preschool children[19]. The CPET data and the reports of EKG and echocardiography of the included participants were reviewed by K-L LIN (who has experience in CPET for more than 15 years) and S-H Tuan (who has experience in CPET for more than 5 years), respectively. Since the preschoolers were all from one medical center, participants with missing data or with outliers in body composition measurement and CPET were excluded to avoid selection bias. This study was approved by the Institutional Review Board of Kaohsiung Veterans General Hospital (number: VGHKS15-CT7-05). All patients provided the informed consent from their parents before the body composition measurement and the exercise testing.

### Cardiopulmonary exercise testing

According to the American College of Sports Medicine (ACSM) guidelines[24] and clinical experiences[25], treadmill exercise testing is possible in children from the age of three. Prior to the CPET, each preschool child in our study was familiarized with the procedures and equipment used in the test via a demonstrative explanation on the different day of the CPET (C-H Yang). The CPET was done under the supervision of one experienced physiatrist (K-L Lin) and a physical therapist (C-H Li). We performed graded symptom-limited exercise testing on a treadmill, equipped with a flow module, a gas analyzer, and an electrocardiographic monitor (Metamax 3B, Cortex Biophysik GmbH Co., Leipzig, Germany), to measure the subjects’ exercise capacity. The testing was also guided by a trained staff (C-H Yang), who stood in the back of the preschoolers (on the frame of the treadmill rather than the walking area), to safeguard the them during the test. We chose Ramped-Bruce protocol under ACSM’s guideline[26]- a protocol that applies continuous steady increase in workload without abrupt changes, since it is commonly and safe to use among children [27].The test was terminated when preschoolers demonstrated subjective unbearable symptoms, when they could no longer continue, or when they attained maximal effort as indicated by the ACSM[28]. VO_2_ and carbon dioxide production (VCO_2_) were measured during the test by using the breath-by-breath method. Blood pressure (BP) and heart rate (HR) during the CPET were also measured. Metabolic equivalent (MET) was calculated after measuring VO_2_.and peak MET was defined as the maximal MET throughout the whole CPET. The anaerobic threshold was determined by VE/VO_2_ and VE/VCO_2_ methods[29]. To be considered a maximal effort the participant must have achieved at least 1 of the following 2 criteria: (1) peak respiratory exchange ratio (RER) > 1.1, and (2) peak heart rate (HR) > 85% of age-predicted maximum [30–32].

The equation VO_2_ = a log (VE) + b was used, and the graphic slope of this equation was determined as OUES[22]. We calculated OUES by the half (OUES-50) and the entire (OUES-100) exercise duration. Given the variable anthropometric changes in children caused by their development, OUES was normalized by the body surface area (BSA) as suggested[21]. The BSA was computed using the Haycock equation: BSA (m^2^) = 0.024265 × Ht^0.3964^ × Wt^0.5378^, where Ht represents the height in centimeters, and Wt refers to the weight in kilograms.

### Anthropometry and body composition

Vector bioelectric impedance analysis (VBIA) was used in this study to measure the body composition of preschoolers. We performed VBIA with the bioelectrical impedance vector analysis software by using the resistance–reactance graph method[33]. Zeus 9.9 PLUS (Jawon Medical Co. Ltd., Kungsang Bukdo, South Korea) was used to analyze the body composition of the preschoolers. The machine sent a minute electric current and measured the body composition by using personal data that have been saved (height, weight, sex, age, and newly calculated body impedance) via the tetrapolar electrode method (electrodes were located on both hands, both soles of the feet, and both ankles of participants with frequencies of 1, 5, 50, 250, 550, and 1,000 kHz and a current of 360 uA). All participants received the anthropometric measures in the morning and before the CPET. The measures were done by the assistance of one well-trained physical therapist (C-H Li). BMI was defined as body weight (kg) divided by height squared (m^2^) while FFMI was defined as FFM (kg) divided by height squared (m^2^).

Excess body adipose was defined as ‘overweight’ and ‘obesity’ by using BMI. The age-and-gender specific reference BMI values suggested by the Ministry of Health and Welfare in Taiwan published in 2013, for normal weight (BMI range: 13.3–16.9), underweight (BMI < 13.3), overweight (BMI ≥ 16.9), and obese (BMI ≥ 18.5) in 4 to 6-year-old boys, and for normal weight (BMI range: 13.1–17.2), underweight (BMI < 13.1), overweight (BMI ≥ 17.2), and obese (BMI ≥ 18.8) in 4 to 6-year-old girls[34].

The 75th–85th percentile for percentage of body fat has proved to correspond with excess adipose in children and adolescents[35], and the 75th percentile for % BF has been used as the criteria for identifying excess adipose in a study of dyslipidemia[36]. To our knowledge, there were no available reference values of FMI and FFMI for Taiwan children, not to mention preschool children. Given this precedent, we defined excess adipose by FMI as a FMI greater than the sex- and age-specific 75th percentile, insufficient adipose by FMI smaller than 5th percentile, and normal adipose by FMI between 5th to 75th percentiles as the suggestion in the study of Weber DR et al. We also defined excess adipose by FFMI as a FFMI smaller than the sex- and age-specific 25^th^ percentile, and normal adipose by FFMI between 25^th^ to 75^th^ percentiles[37].

### Statistical analysis

SPSS for Windows version 19.0 (Released 2010, Armonk, NY: IBM Corp) was used for all analyses. Continuous data were expressed as mean ± SD and categorical variables were presented as absolute numbers or percentages. Normality and homoscedasticity were checked prior to each analysis. The chi-square test was used to test for differences in the distribution between categorized variables except that the difference of percentage of preschool children to achieve maximal effort during exercise testing was done by Fisher’s exact test. The independent t-test was used for normally distributed variables, whereas the Mann–Whitney U test was used for non-normally distributed variables for comparison between (1) preschool girls and boys, and (2) preschool children with normal and excess adipose.

## Results

One hundred and twenty-seven preschoolers were recruited. Since the study aimed to compare the CRF between preschoolers with normal and excess body adipose, preschoolers whose BMI ranged in underweight (n = 4) were excluded. Six preschoolers were excluded by not having EKG examination while 10 were excluded by not having echocardiography data. One preschooler was excluded by having outlier. Finally, data of 106 preschoolers were collected for the final analysis. Table 1 summarizes the baseline characteristics of the 106 participants (boys = 62, girls = 44). The prevalence of overweight and obese preschool girls, boys, and overall by BMI definition were 15.39%, 14.29%, and 14.76%, respectively. Comparison between two genders, girls had significantly higher percentage of body fat, FM, and FMI while boys had higher FFM, and FFMI (all p < 0.05).

**Table 1:** Baseline characteristics of all recruited preschool children.

		Age (years)	Height (cm)	Weight (kg)	BMI (kg/m^2^)	Body fat (%)	N (%)	O (%)	F (%)	FM (kg)	FMI (kg/m^2^)	FFM (kg)	FFMI (kg/m^2^)
Girl	n = 44	5.58 ± 0.64	116.53 ± 4,54	21.81 ± 3.60	15.98 ± 1.89	17.15 ± 6.73	84.09	11.94	3.97	4.01 ± 2.10	2.88 ± 1.45	18.38 ± 1.59	13.35 ± 0.83
Boy	n = 62	5.86 ± 0.43	119.31 ± 6.22	22.85 ± 4.42	15.95 ± 2.28	11.54 ± 5.67	85.48	11.66	2.86	2.88 ± 1.70	1.97 ± 1.17	21.61 ± 3.96	14.64 ± 2.01
Total	N = 106	5.74 ± 0.55	118.13 ± 5.70	22.41 ± 4.09	15.96 ± 2.11	14.41 ± 6.78	84.90	11.78	3.32	3.46 ± 1.98	2.43 ± 1.38	19.96 ± 3.38	13.98 ± 1.64
P value ^a^		0.061	0.102	0.498	0.759	0.009*	0.977			0.039*	0.015*	0.001*	0.003*

BMI: body mass index; N(%), percentage of normal weight subjects; O (%), percentage of overweight subjects; F(%), percentage of obesity subjects; FM, fat mass; FMI, fat mass index; FFM, fat-free mass; FFMI, fat-free mass index

^a^All the comparisons between girls and boys were done by Mann–Whitney U test except p values that marked with ^a^, which was analyzed by independent Chi square test for comparison percentage of excessive adiposity between girls and boys

*p value ≦ 0.05

Table 2 presented the results of comparisons of CRF between participants with excess and normal adipose by BMI, FMI, and FFMI definitions. There were no differences in peak RER and peak HR between preschool children with normal and excess body adipose by all the three definitions. Preschool children with normal adipose had higher OUES-50 and OUES-100 by BMI (p = 0.018 for OUES-50, p = 0.004 for OUES-100) and FFMI (both p = 0.001) definitions. By FMI definition, no significant differences of OUES-50 (p = 0 .062), and OUES-100 (p = 0.285) between preschool children with normal and excess adipose were noted. For comparison of AT MET (p = 0.143, 0.083, 0.898 for BMI, FMI, and FFMI, respectively) and peak MET (p = 0.445, 0.597, 0.590 for BMI, FMI, and FFMI, respectively), preschool children with normal adipose had no significant difference than those with excess adipose by all the three definitions.

**Table 2:** Comparisons of cardiorespiratory fitness between subjects of excess and normal body adipose under different classifications.

		Classify by BMI			Classify by FMI			Classify by FFMI		
		N (n = 90)	E (n = 16)	P value	N (n = 86)	E (n = 20)	P value	N (n = 85)	E (n = 21)	P value
Peak RER		1.16 ± 0.11	1.03 ± 0.07	0.494	1.16 ± 0.12	1.05 ± 0.08	0.507	1.18 ± 0.07	1.05 ± 0.12	0.498
Peak HR		182.67 ± 6.27	176.98 ± 18.25	0.241	183.91 ± 5.09	176.82 ± 12.89	0.154	184.12 ± 8.09	178.16 ± 12.08	0.122
	AT MET	8.18 ± 1.39	7.53 ± 1.13	0.143	8.31 ± 1.27	7.60 ± 1.00	0.083	8.22 ± 1.40	8.11 ± 1.20	0.898
	PEAK MET	10.81 ± 2.04	10.22 ± 1.41	0.445	11.01 ± 2.13	10.39 ± 1.29	0.597	10.92 ± 2.15	10.69 ± 1.37	0.590
	OUES-50	1.19 ± 0.11	1.01 ± 0.27	0.018*	1.22 ± 0.30	1.17 ± 0.12	0.062	1.31 ± 0.22	0.93 ± 0.15	0.001*
	OUES-100	1.25 ± 0.19	1.04 ± 0.23	0.004*	1.25 ± 0.24	1.15 ± 0.25	0.285	1.35 ± 0.29	0.89 ± 0.15	0.001*

BMI, body mass index; FMI, fat mass index; FFMI, fat-free mass index; RER, respiratory exchange ratio; HR, heart rate; N, normal adipose; E, excessive adipose; AT MET, metabolic equivalent at anaerobic threshold; PEAK MET, peak metabolic equivalent during exercise testing; Classify by BMI: normal BMI was defined as normal adiposity, BMI as overweight and obese was defined as excess adipose; Classify by FMI: FMI ≦ 75^th^ percentiles of all the subjects was defined as normal adipose, FMI > 75^th^ percentile of all the subjects was defined as excess adipose; Classify by FFMI: FMI > 25^th^ all the subjects was defined as normal adipose, FFMI ≦25^th^ percentiles all the subjects was defined as excess adipose

*p value ≦ 0.05

Table 3 displayed the comparisons of ability to achieve maximal effort during exercise testing between preschool children with excess and normal body adipose. Among preschool children with normal body adipose, 20.0%, 22.1%, and 16.5% of participants by BMI, FMI, and FFMI classification, respectively, could not perform maximal effort during exercise testing. Meanwhile, among preschool children with excess body adipose, 56.3%, 45.0%, and 47.6% of participants by BMI, FMI, and FFMI classification, respectively, could not meet any criteria of achieving maximal effort. The percentage of preschool children to perform maximal effort during exercise testing showed significant difference by BMI (p = 0.004), FMI (p = 0.043), and FFMI (p = 0.007) classifications.

**Table 3:** Comparisons of ability to achieve maximal effort during exercise testing between preschool children with excess and normal body adipose under different classifications.

Achieving maximal effort during exercise testing^a^		Classify by BMI			Classify by FMI			Classify by FFMI		
		N (N = 90)	E (N = 16)	P value^b^	N (N = 86)	E (N = 20)	P value^b^	N (N = 85)	E (N = 21)	P value^b^
	Not meet	18 (20.0%)	9 (56.3%)	0.004*	19 (22.1%)	9 (45.0%)	0.043*	14 (16.5%)	10 (47.6%)	0.007*
	Meet	72 (80.0%)	7 (43.7%)		67 (77.9%)	11 (55.0%)		71 (83.5%)	11 (52.4%)	

BMI, body mass index; FMI, fat mass index; N, normal adipose; E, excessive adipose; AT MET, metabolic equivalent at anaerobic threshold; PEAK MET, peak metabolic equivalent during exercise testing; Classify by BMI: normal BMI was defined as normal adiposity, BMI as overweight and obese was defined as excess adipose; Classify by FMI: FMI ≦ 75^th^ percentiles of all the subjects was defined as normal adipose, FMI > 75^th^ percentile of all the subjects was defined as excess adipose; Classify by FFMI: FMI > 25^th^ all the subjects was defined as normal adipose, FFMI ≦25^th^ percentiles all the subjects was defined as excess adipose

^a^ To be considered a maximal effort the participant must have achieved at least 1 of the following 2 criteria: (1) peak respiratory exchange ratio (RER) > 1.1, and (2) peak heart rate > 85% of age-predicted maximum.

^b^ The comparison for categorical data was done by Fisher’s exact test.

*p value ≦ 0.05

## Discussion

In this study, we found that preschool children with excess body adipose by both BMI and FFMI classifications showed significantly poorer ability to perform maximal effort during exercise testing. We also observed that preschool children with excess body adipose had lower OUES-50 and OUES-100 (but not AT MET or peak MET) than those with normal body adipose no matter in BMI or FFMI classifications. Moreover, the similar results could not be observed while comparing the two groups by FMI classification.

Studies had proved that school children aged more than 6 years old attain and maintain maximal effort in exercise testing poorly than adults since they did not regularly engage in strenuous exercise and their bodies were not adapted to that [21, 38]. In our study, we observed that even in preschool children with normal body adipose, there was around 20% (no matter by which classifications) participants could not reach maximal effort during exercise testing. Few studies in children aged less than 7 years old had proved that they had poorer ability to perform maximal effort as similar as out results[19, 39]. What is more, we also found that preschool children with excess body adipose had significant lower percentage to achieve maximal effort than those with normal body adipose by BMI and FFMI classifications. This might indicate that excess body adipose has negative effect on maintain and sustain during strenuous exercise, a finding that was similar to the one by BreithaupT G. et al, who evaluated the CRF in obese children aged 7 to 18 years old[40]. In addition, since we used Ramped-Bruce protocol with treadmill in this study, the increased energetic costs of PA in preschool children with excess body adipose could potentially affect performance during an incremental test, with exhaustion occurring prior to attainment of $\dot{V}\mathrm{O2max}$.

OUES has been proved an objective and noninvasive CPET parameter that requires no maximal effort. It may be indicative of cardiorespiratory fitness during exercise in healthy children aged more than seven years old[21, 41]. OUES-100 has been proved to correlate strongly with peak O_2_ in healthy children aged more than seven years old[41]. It our past study, we found that OUES correlated positive-moderately with peak MET and peak VO2 in preschool children older than 4 years old[19]. Many studies had shown that health-related physical fitness is associated with total and central body fat among preschoolers[18, 42] but little available studies assessed preschoolers’ CRF by CPET. In this study, we observed that preschool children with excess body adipose had lower OUES-50 and OUES-100 than those with normal body adipose no matter in BMI or FFMI classifications. This finding coincided with the study by Henriksson et al, who showed that preschool children (aged 4.48 ± 0.15 years old) with higher FFMI was associated with better CRF (determined by PREFIT-20 m shuttle-run test)[18].

In contrast to OUES, no significant difference in peak MET was noted between preschool children with normal and excess body adipose by all the three classifications. This finding was contradictory to studies in school children and adolescents, which proved that those with excess body adipose had significantly lower peak maximal oxygen consumption or peak MET[43, 44]. Indeed, to carry out determinations of VO_2_ max in children (not to mention preschool children and children with excess body adipose), is difficult due to many different factors, like lack of physical fitness and motivation, uncomfortable while using mask to collect samples of expired air and so on[45]. Therefore, many studies comparing the results of incremental CPET and supramaximal constant load verification CPET have been conducted, though with controversial conclusions[46]. Since we also found that relatively larger numbers of preschool children, whether in normal or excess body adipose group, could not reach maximal effort, we could suspect that the peak MET might not reflect the maximal effort truly in preschool children, and thus resulted in these findings in respect of peak MET.

In inconsistent with our presumption that preschool children with excess body adipose by FMI classification might have lower CRF, we didn’t observe significant difference between preschool children with normal and excess body adipose no matter in respect of peak MET or OUES. A higher FMI has been reported in healthy and normal functioning adults (‘healthy obese’) while those suffering from muscle wasting can present with a preserved FMI, but in reality with lower FFMI[47]. It’s not only body adipose in anthropometry but also skeletal muscle mass, waist circumference, and so on might affect CRF[42]. Therefore, four typical situations had been stated for anthropometric assessment in adolescents: low FFMI and high FMI (corresponding to obesity); low FFMI and low FMI (corresponding to leanness); high FFMI and low FMI (corresponding to muscle hypertrophy); and high FFMI and high FMI, (corresponding to combined excess of FFMI and FMI)[48]. However, the reference values of FMI and FFMI among preschool children in Taiwan was not established before this publication, and the subgroup analysis by these two indices couldn’t do in this current study.

This study featured the following limitations. First, since this was a cross-sectional study, it should be interpreted carefully the results regarding the relationship between body adipose and CRF levels in preschoolers of Taiwan. Conclusions on the direction of the relationships cannot be drawn. Moreover, since this was an observational study, we did not have the data about potential confounders that might be associated with body adipose and CRF, such as socioeconomic status of family, waist circumference and so on. Second, since there was no available survey of normal distribution of FMI and FFMI data among Taiwanese preschool children before the publication of this study, it is unclear whether preschoolers in this study could represent the whole Taiwanese preschool population. In addition, to check the generalization of our data, we did literature review to find reference values of FMI and FFMI in preschoolers in countries where people have the similar stature and eating habit with Taiwan. One available norms of FMI and FFMI of Japan in young children aged between 3 and 5 years[49] were compared to our data and both the two studies showed similar trends of FMI and FFMI. Lastly, the sample numbers in preschoolers with excessive adipose were relatively small although the statistical power was sufficient. Moreover, the participants were only recruited in one medical center. The results might not be applied to whole nation even though the prevalence of overweight and obese preschool children was similar to data found in the national survey in Taiwan[34]. At last, body composition might differ between sex and change as aging. Due to small numbers, we did not do a subgroup analysis in respect of different genders.

To the best of our knowledge, this study was the first to compare the CRF measuring directly by CPET between preschool children with normal and excessive body adipose. In this study, we demonstrated that though preschool children with excess body adipose by both BMI and FFMI classifications had poorer ability to perform maximal effort than those with normal body adipose, preschool children no matter with normal or excessive body adipose were more difficult to achieve maximal effort during exercise testing than older children. Preschool children with excessive body adipose had lower OUES-50 and OUES-100 than peers with normal body adipose in both BMI and FFMI classifications. Childhood obesity is associated with a higher risk of adult obesity, as well as impaired health later in life[18]. Higher CRF in childhood and adolescence have been associated with a healthier cardiovascular profile later in life and with a lower risk of premature death[50]. Our results emphasized that those findings in school children might be the implicated in preschool children and weight control and health promotion should be encouraged as early as possible.

## Conclusion

Our study demonstrated that preschool children with excess body adipose by both BMI and FFMI classifications had poorer ability to perform maximal effort during exercise testing. They also had lower OUES-50 and OUES-100 than peers with normal body adipose no matter by BMI or FFMI classifications. Excess body adipose was in negative association with the CRF of preschool children. Therefore, weight control and health promotion should start as early as possible. Future larger cross-national studies to provide reference values of FMI and FFMI and to assess the association between CRF and body adipose among Taiwanese preschool children are warranted.

## Abbreviations

PA: physical activity
BMI: body mass index
CRF: cardiopulmonary fitness
FM: fat mass
FFM: fat-free mass
%FM: percentage of fat mass
FMI: fat mass index
FFMI: fat-free mass index
CPET: cardiopulmonary exercise testing
OUES: oxygen uptake efficiency slope
VO2: oxygen consumption
ACSM: American College of Sports Medicine
VCO2: carbon dioxide production
BP: blood pressure
HR: heart rate
MET: metabolic equivalent
RER: respiratory exchange ratio
OUES-50: OUES calculated by half of the CPET duration
OUES-100: OUES calculated by the whole CPET duration
BSA: body surface area
VBIA: vector bioelectric impedance analysis
